# Identification of Gene-Set Signature in Early-Stage Hepatocellular Carcinoma and Relevant Immune Characteristics

**DOI:** 10.3389/fonc.2021.740484

**Published:** 2021-10-22

**Authors:** Qijie Zhao, Rawiwan Wongpoomchai, Arpamas Chariyakornkul, Zhangang Xiao, Chalermchai Pilapong

**Affiliations:** ^1^ Center of Excellence for Molecular Imaging (CEMI), Department of Radiologic Technology, Faculty of Associated Medical Sciences, Chiang Mai University, Chiang Mai, Thailand; ^2^ Department of Pathophysiology, College of Basic Medical Science, Southwest Medical University, Luzhou, China; ^3^ Department of Biochemistry, Faculty of Medicine, Chiang Mai University, Chiang Mai, Thailand; ^4^ Laboratory of Molecular Pharmacology, Department of Pharmacology, School of Pharmacy, Southwest Medical University, Luzhou, China; ^5^ South Sichuan Institute of Translational Medicine, Southwest Medical University, Luzhou, China

**Keywords:** early-stage hepatocellular carcinoma (eHCC), immune cells, PRKDC, prognosis, gene-set signature

## Abstract

**Background:**

The incidence of hepatocellular carcinoma (HCC) is rising worldwide, and there is limited therapeutic efficacy due to tumor microenvironment heterogeneity and difficulty in early-stage screening. This study aimed to develop and validate a gene set-based signature for early-stage HCC (eHCC) patients and further explored specific marker dysregulation mechanisms as well as immune characteristics.

**Methods:**

We performed an integrated bioinformatics analysis of genomic, transcriptomic, and clinical data with three independent cohorts. We systematically reviewed the crosstalk between specific genes, tumor prognosis, immune characteristics, and biological function in the different pathological stage samples. Univariate and multivariate survival analyses were performed in The Cancer Genome Atlas (TCGA) patients with survival data. Diethylnitrosamine (DEN)-induced HCC in Wistar rats was employed to verify the reliability of the predictions.

**Results:**

We identified a Cluster gene that potentially segregates patients with eHCC from non-tumor, through integrated analysis of expression, overall survival, immune cell characteristics, and biology function landscapes. Immune infiltration analysis showed that lower infiltration of specific immune cells may be responsible for significantly worse prognosis in HCC (hazard ratio, 1.691; 95% CI: 1.171–2.441; *p* = 0.012), such as CD8 Tem and cytotoxic T cells (CTLs) in eHCC. Our results identified that Cluster C1 signature presented a high accuracy in predicting CD8 Tem and CTL immune cells (receiver operating characteristic (ROC) = 0.647) and cancerization (ROC = 0.946) in liver. As a central member of Cluster C1, overexpressed PRKDC was associated with the higher genetic alteration in eHCC than advanced-stage HCC (aHCC), which was also connected to immune cell-related poor prognosis. Finally, the predictive outcome of Cluster C1 and PRKDC alteration in DEN-induced eHCC rats was also confirmed.

**Conclusions:**

As a tumor prognosis-relevant gene set-based signature, Cluster C1 showed an effective approach to predict cancerization of eHCC and its related immune characteristics with considerable clinical value.

## Introduction

Liver cancer is the fourth leading cause of cancer-related deaths worldwide (1). Hepatocellular carcinoma (HCC) accounts for more than 90% of liver cancers and is non-negligible reason of most patients’ death (2). Over the past 20 years, the detection of patients with HCC was increased, and surgical resection obviously ameliorated the 5-year overall survival (OS) (3), while even in these cases, the high recurrence ratio and no effective adjuvant therapies presently available are common (4). Moreover, the metastasis strength is responsible for most HCC-associated morbidity and mortality (5, 6). Investigation of molecular and systematic mechanisms of HCC may be useful to predict early-stage HCC (eHCC) and prevent the progress to advanced stage. The HCC tumorigenesis and metastasis are multistep processes and are known to be regulated by tumor immune microenvironment (7, 8). Although immune disorder in HCC has been well studied (9, 10), the dysregulated tumor immune microenvironment in eHCC is still far from clarified, especially in immune cell conditions. A better understanding of how specific cellular tumor transcriptome functions contribute to HCC stratification and specific tumor microenvironment (TME) is needed to enable customized treatment design and novel immunotherapy exploitation.

Oncogene-driven immune mediators allow tumor cells to immune evasion and thrive in the TME (11). Most studies of HCC showed that oncogene expression is associated with patients’ OS, somatic driver mutations, and abnormal immune cells (12, 13), but whether heterogeneity in different subtypes of HCC can be stratified by gene set-based signature has not been well established. Furthermore, genetic alteration-related gene expression plays an important role in HCC formation, which was also significantly higher in eHCC (14). Studies have shown that the treatment response and survival outcome of HCC patients not merely depend on tumor stage but also are associated with TME heterogeneous and molecular features (15–18). Strategies to identify the subset of HCC likely to have different transcriptome and immune characteristics are important for diagnosis and additional clinical therapy (19–21). Biomarkers, especially gene expression in tumor tissues, are reliably related to HCC prognosis and TME characteristics (22, 23). Recently, the higher mutation of PRKDC has been regarded as a new target for checkpoint blockade immunotherapy, which was identified as one of the top most frequently mutated DNA repair genes in liver cancer (24). In addition, knockout PRKDC has shown the ability to enhance anti-PD-1 antibody treatment in tumor models (24). Therefore, the further analysis based on large and comprehensive datasets in combination with more potential markers may provide an opportunity to identify signature for eHCC and to improve personalized medicine.

The TME context, consisting of heterogeneous populations including tumor cells themselves, infiltrating immune cells, and secreted factors, has been reported to highly associate with tumor progression, prognosis, and therapeutic responses (18, 25, 26). The interaction between tumor cells and immune cells was gradually recognized and then updated into the emerging hallmarks of tumor until 2011 (27). In the liver, it is important to distinguish between the TME of eHCC, a common condition in primary HCC, and the TME of non-tumors. The TME components based on computational evaluation have been utilized to predict cancer prognosis and design more effective therapeutic strategy (28–30), which also connects with tumor subtype stratification (31). Recently, Zeng et al. established a comprehensive TME model as a prognostic biomarker and immunotherapeutic benefit indicator of stomach cancer (32). To date, the comprehensive landscape of TME-related gene set-based signatures in the eHCC has not yet been elucidated.

To address these issues, we stratify HCC according to clinical stage and integrated multiple cohorts with gene expression data to develop and validate individualized gene set-based survival, and mutational and gene expression signature for eHCC. Furthermore, the relationship between stratified HCC and the TME immune characteristics was estimated to investigate the immune-disorder mechanisms and therapeutic targets. Finally, we applied eHCC rat models for experimental verification to prove the stability and reliability of gene-set predictive value and potential target.

## Materials and Methods

### Samples and Clinical Data Description

We systematically searched for HCC gene expression that were publicly available and reported with pathological stage annotations. We downloaded the publicly available expression data for filtering and analysis. In total, 18 eligible HCC cohorts were divided into three groups according to the three different expression platforms, as The Cancer Genome Atlas (TCGA) and Genotype-Tissue Expression (GTEx) (bulk RNAseq data), Affymetrix Human Genome U219, and Affymetrix Human Genome U133. We downloaded the raw array Affymetrix^®^ “CEL” files (**Table S7**) from the Gene Expression Omnibus (GEO) accession viewer and adopted a robust multiarray averaging method with the affy package default parameters to perform background adjustment and quantile normalization. Gene expression values of all probes were adjusted by dplyr software in each dataset. To identify the risk of HCC development and most likely to suffer from genetic dysregulation, we defined the eHCC (Bclc 0-A or early marker) and advanced-stage aHCC (Bclc B-C or advanced marker) patients in GEO dataset. As to datasets in TCGA and GTEx, TCGA tumor and GTEx non-tumor sample RNAseq expression data (transcripts per million reads (TPM) value) were downloaded from the UCSC Xena browser; the data were extracted and preprocessed with Toil workflow software in default parameters [a reproducible, open-source scientific workflow for big biomedical data analysis in UCSC (33)]. Toil pipeline uses the single script to compute gene- and isoform-level expression in multiple platforms, which can efficiently decrease the batch effect with the normalized TPM value. The baseline information of each eligible ESCA data was obtained from TCGA, such as available follow-up time and pathological stages. The eHCC (Stage I–II) and aHCC (Stage III–IV) from TCGA dataset were used in the current study. The batch effects between different datasets within the same platform were adjusted by ComBat algorithm (34). Then, we use three different platform data for analysis: 1) TCGA and GTEx; 2) Affymetrix Human Genome U219 platform: GSE63898; and 3) Affymetrix Human Genome U133 platform: GSE101685, GSE45436, GSE6222, GSE62232, GSE6764, GSE9843, GSE102079, GSE121248, GSE49516, GSE112790, GSE19665, GSE29721, GSE45267, GSE58208, GSE84402, and GSE88839.

### Somatic Mutation and Copy Number Variation

The somatic mutation data (MuTect2) of TCGA LIHC patients were also achieved from TCGA dataset (https://portal.gdc.cancer.gov/) and summarized using maftools (35). For each gene, the mutation frequency in corresponding eHCC patients was ranked in order. The LIHC dataset from Affymetrix SNP 6.0 was applied for individual copy number variation (CNV) analysis. The sequence data for the cis-expression quantitative trait locus (cis-eQTL) study was filtered based on somatic mutation files, and forward stepwise conditional analysis implemented in MatrixEQTL was conducted (36).

### Unsupervised Clustering for Prognostic Subtypes

“Favorable prognostic genes” (n = 15) and “Poor prognostic genes” (n = 38) were used as favorable prognosis genes and poor prognosis genes, respectively. Consensus clustering was evaluated on favorable prognosis and poor prognosis genes with ConsensusClusterPlus (parameters: reps = 100, pItem = 0.8, pFeature = 1) (37). Ward.D2 and Spearman’s distances were used for clustering algorithm and distance metric, respectively. With selected Clusters C1 and C3, median expression levels of co-expressed favorable prognosis and poor prognosis genes were used to assign quiescent (Cluster C1 ≤ 0, Cluster C3 ≤ 0), poor prognosis (Cluster C1 > 0, Cluster C3 ≤ 0), favorable prognosis (Cluster C1 ≤ 0, Cluster C3 > 0), and mixed (Cluster C1 > 0, Cluster C3 > 0) subgroups to each sample. For each subgroup, each of the sample was tested, computing a Fisher’s exact test to determine whether Cluster C1/C3-based stratification functions were significant in eHCC and aHCC patients.

### Generation of Immune Cell Infiltration

We partially established a predictive immune infiltration pattern from the immune cells metagenes, which were combined with the sources reported by Ru et al. (38) and Bindea et al. (39). The selected immune cell metagene includes 15 categories according to T cell-related immune cells, such as regulatory T cells (Tregs), dendritic cells (DCs), and subtypes of T cells. To quantify the proportions of immune cells in the HCC samples, we used the single-sample gene-set enrichment analysis (ssGSEA) algorithm to evaluate the relative abundance of each cell infiltration from three independent cohorts.

### Correlation Between Cluster Gene Signature and Other Related Biological Processes

For crosstalk analysis of the different elements in HCC, we integrated the Cluster gene signatures to further investigate its function in subtypes of HCC, and we termed it as signature score. The expression of each gene in the Clusters was first transformed into a z-score. Then, a principal component analysis (PCA) was used to predict selected Cluster gene signature, and principal component 1 was extracted to serve as the signature score. This approach has the advantage of focusing the score on the set with the largest block of well-correlated (or anticorrelated) genes in the set while down-weighting contributions from genes that do not track with other set members. Subsequently, the estimated signature score was used to infer the correlation between different clusters and immune cell infiltration in subtypes of HCC. The correlation coefficients were computed by Pearson’s test.

### Construction of Overall Survival and Prognostic Signature

After removal of the patients without complete clinical information in TCGA, 365 samples with complete OS information were finally obtained and used for further analysis. Survival analysis associated with selected differentially expressed genes (DEGs) was performed by the Kaplan–Meier analysis, and the cutoff point of each dataset subgroup was determined using the survminer R package. The “surv-cutpoint” function, which iteratively tested all possible cut points in order to find the maximum rank statistic, was adopted to dichotomize patients into low- and high-risk groups based on the maximally selected log-rank statistics to decrease the batch effect of calculation (threshold filtering >30%). Meanwhile, according to cluster subgroups, pathological stage, and immune infiltration, patients were divided into multiple groups. The multivariate survival curves for the above groups were generated *via* the Kaplan–Meier method and log-rank tests to determine significance of differences. Moreover, based on “surv-cutpoint” function, we obtained immune cells related a higher-risk group with maximum rank statistic for poor prognostic signature analysis. The poor prognostic signature frequencies were calculated by maximum rank statistic in both tumor and non-tumor samples.

### Functional and Pathway Enrichment Analysis

The Gene Ontology (GO) function (40) and Kyoto Encyclopedia of Genes and Genomes (KEGG) pathway (41) enrichment regarding the DEG expression in different stratifications of HCC were analyzed using the Clusterprofiler R package (42). The GO function including biological process (BP), molecular function (MF), and cellular component (CC). To explore the underlying function between high- and low-immune infiltration groups, GSEA (http://software.broadinstitute.org/gsea/index.jsp) was implemented to determine the enrichment of a certain gene rank in the pre-defined BPs. *p* < 0.05 was chosen as the cutoff criterion. A developing R package enrichplot (https://github.com/GuangchuangYu/enrichplot) implements several visualization methods to help in interpreting enrichment results and was adopted to visualize immune-relevant gene clusters. Furthermore, we measure the functional similarity among Cluster C1 proteins by ranking their average value inside the interactome. Functional similarity, which is defined as the geometric mean of their semantic similarities in BP, MF, and CC aspects of GO, is designed for measuring the strength of the relationship between each protein and its partners by GOSemSim package (43) using the Wang method.

### Protein–Protein Interaction Network Analysis

The genomic association between Cluster C1 genes and PRKDC was querying in STRING (44) and exploring their relevant network, which was based on retrieval of interacting Genes/Proteins. The combined score was generated from co-expression, experimentally determined interaction, homology, database annotation, and automated textmining.

### Animal Model

Five-week-old male Wistar rats (Nomura Siam International, Bangkok, Thailand) were housed and acclimated in specific pathogen-free cages of laboratory animal center, Chiang Mai University, under a 12-h light/dark cycle at 21°C ± 1°C and 50% ± 10% humidity. All animals had free access to food and water. Quality of life of all animals was monitored during the experiments according to the suggestion of the animal ethical committee. For construction of HCC model, rats were intraperitoneally injected with diethylnitrosamine (DEN; Sigma) at 50 mg/kg (b.w.) once a week and were continuously housed without DEN induction for 4 weeks (defined as eHCC) and for 8 weeks (defined as aHCC). For healthy rats, the rats were intraperitoneally injected with normal saline (4 ml/kg, b.w.) once a week for 4 weeks and were continuously housed without any induction for 8 weeks. At the time of sacrifice, rats were anesthetized using isoflurane, and liver tissues were collected for histological analysis and RNA sequencing. Animal experiment has been approval by Animal Ethics Committee of Chiang Mai University.

To verify our prediction in such HCC rat model, two rats per group (normal, eHCC, and aHCC) were selected to perform RNA sequencing. Selection criteria for eHCC and aHCC initially relied on gross appearance of tumor nodules in the liver at the time of sacrifice. Livers of DEN-induced rats without clear appearance of tumor nodule was chosen as the eHCC model, while that having several tumor nodules was chosen as the aHCC model. In addition, H&E staining was also conducted to investigate morphological change in each group.

### RNA Isolation and Library Preparation

Total RNA from liver tissue of normal, eHCC, and aHCC rats was isolated using NucleoSpin RNA Plus kit (Macherey-Nagel, Catalog no. 740984.50). The quality and integrity of total RNA were checked by an Agilent Bioanalyzer 2100 system and agarose gel electrophoresis. After a quality control (QC) procedure was performed to check quality and integrity of total RNA, mRNA was purified using poly-T oligo-attached magnetic beads; and the cDNA libraries were constructed, according to manufacturer’s recommendations (Novogene Corporation, Beijing, China). All libraries were sequenced using the Illumina HiSeq PE150 platform bp. The library construction and sequencing were performed by the Novogene Corporation. The raw RNAseq data were first processed by the Hisat2 software (default parameters) to remove the rRNA contamination and filter the user-specified adaptor sequences by Python. The purified data were used for QC tool (tmkQC.py) with both quality check (base threshold >20, proportion of low-quality bases in reads <10%) and data processing capability. Then, the high-quality and clean reads were aligned (mm10 mouse reference) with UCSC assembly and aligned by Hisat2 software with default parameters. Raw read counts for rat model were assigned to gencode.vM23 genes. The gene expression values were fragments per kilobase of exon per million mapped fragments (FPKM) normalized by htseq-counts software and converted to TPM.

### Statistical Analysis

All statistical analyses were performed using R (version 4.0.2) with several publicly available packages and GraphPad Prism 8.0. The Kruskal–Wallis tests were used to conduct difference comparisons of three or more groups (45). IGV software was used for sequencing data visualization (46). Correlation coefficients between the expression of genes were computed by Pearson’s and distance correlation analyses. The package pROC (47) was used to construct receiver operating characteristic (ROC) curves to ascertain the area under the curve (AUC) and confidence intervals to estimate the diagnostic accuracy of specific genes in eHCC and immune characteristics. *p*-Values of less than 0.05 were considered statistically significant, and the *p*-values were two-sided.

## Results

### Development and Identification of the Specific Early-Stage Hepatocellular Carcinoma Gene Set in The Cancer Genome Atlas and Gene Expression Omnibus Cohorts

**Figure 1:**
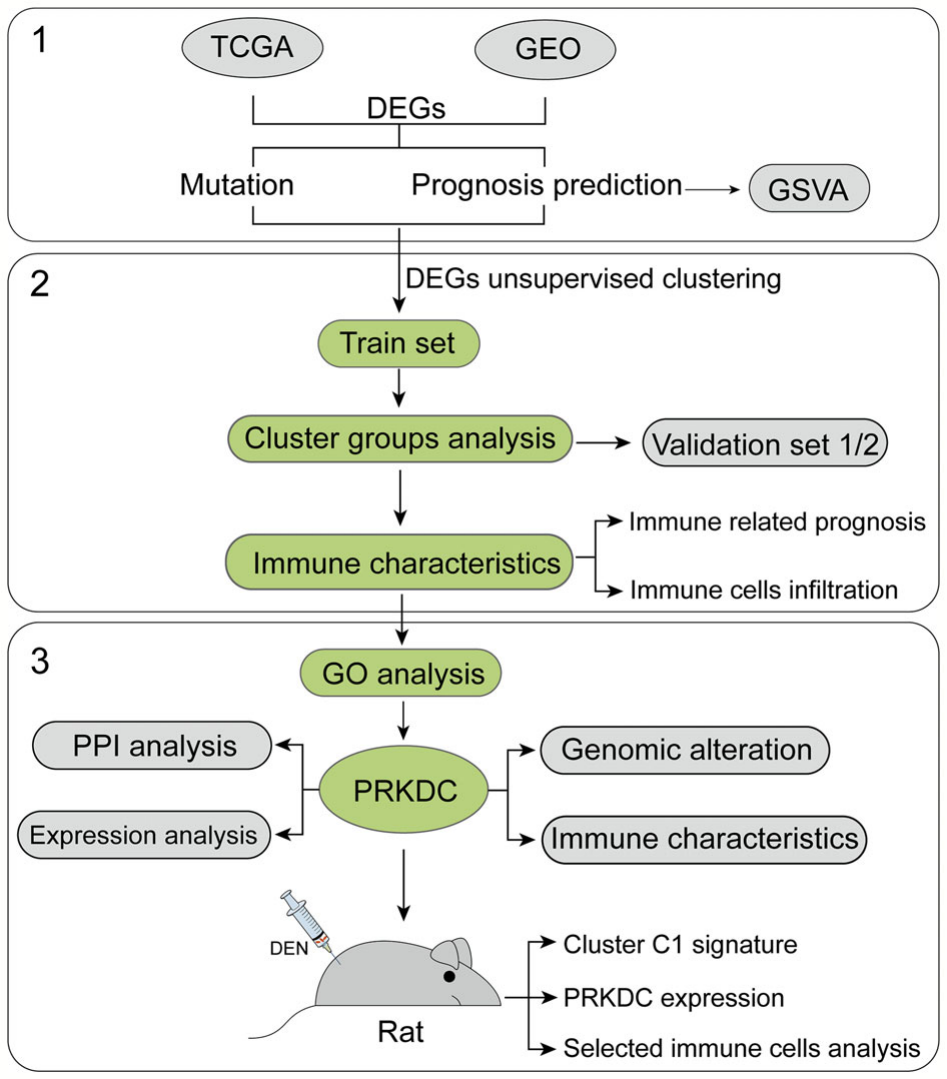
Flowchart of the study. Nineteen public HCC datasets containing 1,408 tumor and non-tumor cases were included and categorized into three independent cohorts according to the data platform. We developed the eHCC-related gene-set signature in the training set and two validation sets. Further, we integrated gene-set signature with immune characteristics, prognosis, genic alteration, and biological functions to investigate the prognostic value. HCC, hepatocellular carcinoma; eHCC, early-stage hepatocellular carcinoma.

At present study, we managed three major steps to establish accurate and reliable gene-set signature for eHCC (**Figure 1**). First, DEGs in both TCGA (train set) and GEO (validation set) were filtered and integrated to conform those with higher mutation frequency and significant prognosis. Then, selected DEGs were clustered into three groups in the train set and validated in the two independent validation sets. The significant cluster group was further used for immune characteristics analysis. Next, through the GO function analysis, we identified the selected signature genes biological functions and interactions. The core marker PRKDC-related expression, immune characteristics, and genic alteration were also analyzed. Finally, we construct HCC rat model; the sequencing data were used for the validation of the signature and PRKDC functions and related characteristics. The immune cell characteristics from different subtypes of HCC and connection to the specific gene set-based signature were investigated. A total 879 patients with HCC and 529 non-tumor samples from 19 independent gene expression datasets with available clinical information were applied for the study (**Table S1**). DEN-induced HCC rat model was constructed to verify the reliability of the predictions.

**Figure 2:**
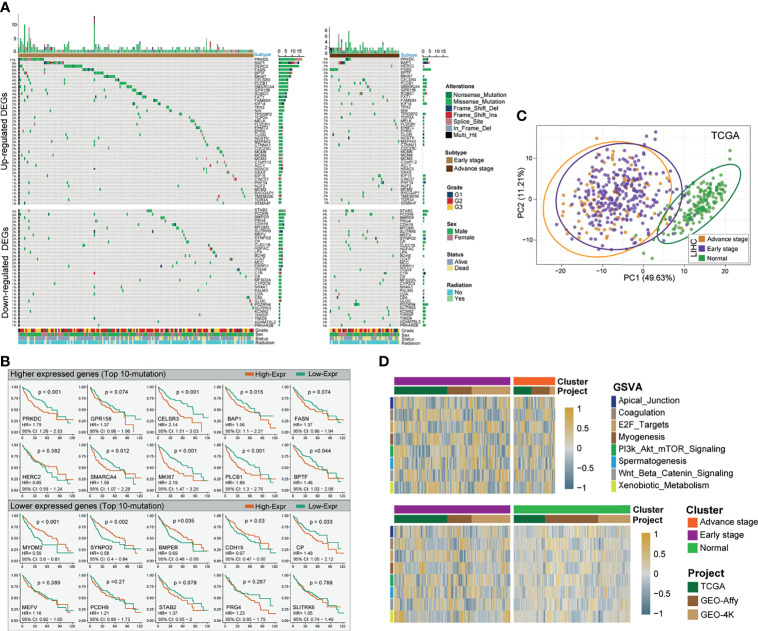
Identification of differentially expressed HCC-related genes and prognostic signature. **(A)** Mutational landscape of significant upregulated/downregulated DEGs of HCC patients with two clinical pathological stages (left profile, eHCC; right profile, aHCC). The right panel is mutation frequency. The top panel shows mutation load of each patients. The middle panel depicts mutation types color coded differently. The bottom panel displays clinical features such as tumor grade, radiation, gender, and vital status. **(B)** Kaplan–Meier survival analyses of selective mutational DEGs. Top 10 mutational DEGs are shown in the graph, the top panel shows upregulated DEGs, and bottom panel show downregulated DEGs. Patients are stratified into low (orange) and high (green) immune risk groups with a cutoff of the maximum value from survminer package. Patients are stratified into low-risk (red) and high-risk (blue) groups with a cutoff of the maximum statistic value in TCGA. **(C)** Principal component analysis (PCA) for the transcriptome profiles of significant prognostic DEG patterns, including eHCC, aHCC, and non-tumor groups. **(D)** GSVA enrichment analysis showing the activation states of specific DEG biological process in distinct subgroups of HCC. The heatmap was used to visualize these biological processes; and the brown color represents activated processes, and navy blue represents inhibited processes. The three independent cohorts were used as sample annotations. Top: eHCC vs. aHCC. Bottom: eHCC vs. non-tumor. HCC, hepatocellular carcinoma; DEG, differentially expressed gene; eHCC, early-stage hepatocellular carcinoma; aHCC, advanced-stage hepatocellular carcinoma; TCGA, The Cancer Genome Atlas; GSVA, gene-set variation analysis.

By investigating TCGA and GEO patients’ mRNA expression, three platform cohorts—1) TCGA&GTEx, 2) Affymetrix Human Genome U219, and 3) Affymetrix Human Genome U133 DEGs—were obtained, independently. After the three independent cohorts of DEGs were integrated and overlapped, 686 DEGs were obtained, among which 414 genes were upregulated and 272 downregulated (**Table S2**). The significantly altered gene expression between HCC and non-tumor tissues across the three independent cohorts was interactively compared using the limma package. The detectable DEGs were identified with the cutoffs |Log fold-change| > 1 and Benjamini–Hochberg-adjusted *p* < 0.05. Somatic mutational profiles of 166 eHCC and 49 aHCC patients from TCGA were analyzed. Overall, the overexpressed and lower-expressed DEGs between two group were presented in rank order (partly data not shown) (**Figure 2A**). Apart from well-known driver oncogenes, we identified the selected DEGs that possessed a significantly altered somatic tumor mutation load in eHCC. Compared with aHCC, upregulated-DEG PRKDC (11% vs. 8%), HERC2 (9% vs. 2%), BPTF (6% vs. 0%), MKI67 (5% vs. 2%), and MCMs (2% vs. 0%) were found to have a higher frequency of somatic mutations in the eHCC patients, whereas downregulated-DEG mutation frequencies were notably lower in eHCC patients, such as STAB2 (3% vs. 8%), PCDH9 (3% vs. 6%), SUTRPK6 (2% vs. 4%), and MEFV (2% vs. 4%). The relationship between the tumor driver mutation and different stages revealed that mutation burden was most close to upregulated DEGs.

For commonly altered genes in eHCC, the survival analysis *via* the Kaplan–Meier method revealed that the expression levels of 53 DEGs were significantly associated with prognosis in TCGA HCC. Among which, the polarized prognostic risk signature between two types of DEGs was concurrent (**Figure 2B**), including 38 poor prognosis indicators (upregulated DEGs) and 15 favorable prognosis indicators (downregulated DEGs) (**Table S3**). The PCA utilizing all prognostic risk signature DEGs reveals clear separation for eHCC/aHCC and non-tumor groups (**Figure 2C**). However, the same trend was not observed for HCC patients in early and advanced stages. To explore the biological behaviors among these distinct DEG patterns, we performed gene-set variation analysis (GSVA) enrichment analysis between three groups. As shown in **Figure 2D** and **Table S4**, these were markedly enriched in cell cycling carcinogenic activation pathways such as E2F-target, PI3K/AKT/mTOR pathway, and Wnt/Beta-Catenin pathway (48–50).

### Dual Analysis of Prognostic Indicator Gene Expression Identifies Subgroup Function of Hepatocellular Carcinoma

**Figure 3:**
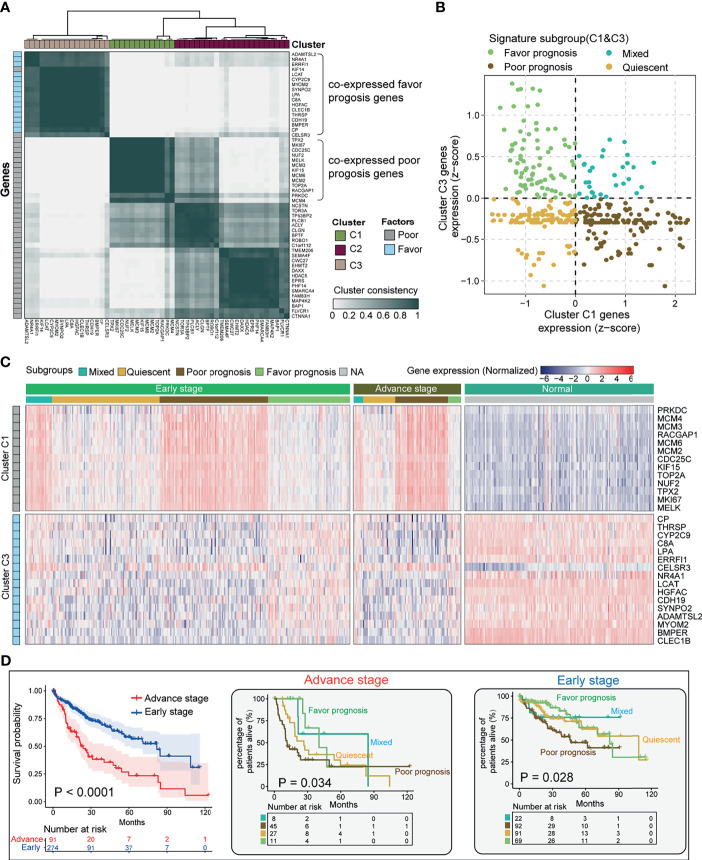
Stratification of HCC tumors based on significant prognostic DEGs. **(A)** Heatmap depicting consensus clustering solution (k = 3) for significant prognostic DEGs (poor/favorable) in the advanced and early diagnostic HCC patients (n = 365). **(B)** Scatter plot showing median expression levels of Cluster C1 (x-axis) gene and Cluster C3 gene (y-axis) in each HCC patient. Signature subgroups were assigned based on the relative expression levels of selected Cluster genes. **(C)** Heatmap depicting expression levels of Cluster C1 and Cluster C3 genes across each subgroup in different pathological stages. **(D)** Kaplan–Meier survival analysis of early-stage and advanced-stage HCC patients (left). Multiple survival curve analysis of advanced-stage (med) and early-stage (right) HCC divided by signature subgroup Cluster C1. Log-rank test *p*-values are shown. HCC, hepatocellular carcinoma; DEG, differentially expressed gene.

In order to stratify eHCC and aHCC based on these prognostic indicator gene expression levels, we utilized transcriptome data from TCGA and validated in GEO database. To enrich for tumor-specific mRNA, filtering was performed to exclude the non-tumor samples in three cohorts. Genes belonging to reactome gene sets “upregulated DEGs” (n = 38) and “downregulated DEGs” (n = 15) were selected for analysis. Previous studies have demonstrated HCC heterogeneity in gene expression, including metastasis, relapse, and prognosis, between biologically distinctive tumor types (51, 52). To aid in selecting genes co-regulated within each group and relevant to subtypes of HCC, we applied consensus clustering to identify two groups of robustly co-expressed upregulated DEGs (Cluster C1; n = 13) and downregulated DEGs (Cluster C3; n = 15) to be used for eHCC evaluation in TCGA (**Figure 3A**). The unsupervised cluster results showed a concordance in both TCGA (**Figure 3A**) and GEO datasets (**Figure S1**). In TCGA, the median expression of Cluster C1 and Cluster C3 genes was calculated for each sample and used in assigning one of four prognostic signature profiles associated with these to cluster subtypes: quiescent, poor prognosis, favorable prognosis, and mixed (**Figure 3B**). Expression levels of Cluster C1 and Cluster C3 genes across the subgroups are presented in **Figure 3C**, including non-tumor samples. The poor prognosis phenotype defined the largest group of cases (137/365; 37.5%), over than mixed (30/365; 8.2%), quiescent (118/365; 32.3%), and favorable prognoses (80/365; 21.9%) in TCGA dataset. Moreover, the proportion of samples belonging to each subtype was statistically significant (Fisher’s exact test *p* = 0.016) between aHCC and eHCC samples (**Table 1**), and the poor prognosis phenotype of aHCC (45/91; 49.5%) is obviously higher than that of eHCC (92/274; 33.5%).

**Table 1:** The clusters defined phenotype in HCC stratification.

Stages	Poor prognosis	Mixed	Quiescent	Favorable prognosis	*χ* ^2^	*p*-Value
**eHCC**	92	22	91	69	10.24	0.016
**aHCC**	45	8	27	11		

HCC, hepatocellular carcinoma; eHCC, early-stage HCC; aHCC, advanced-stage HCC.

In order to determine if Cluster C1- and Cluster C3-based DEGs have an impact on different subgroups of HCC, we performed multi-Kaplan–Meier survival curves using the identified four phenotypes in TCGA eHCC (log-rank test; *p* = 0.028) and aHCC (log-rank test; *p* = 0.034) (**Figure 3D**). Notably, a poor survival outcome was observed in cases with aHCC associated with the shorter median survival (log-rank test; *p* < 0.0001, left). Moreover, Cluster C1 determined that poor prognosis cases had the shortest median OS in both eHCC and aHCC subgroups (**Figure 3D**, right panel). Meanwhile, Cluster C1 contributed to a worse OS in aHCC compared with eHCC [hazard ratio (HR) = 2.943, *p* = 0. 0001]. In addition, quiescent cases had the shorter OS in aHCC (HR = 1.756, *p* = 0.2873 vs. mixed), while mixed cases showed a poor outcome than quiescent in the eHCC group (HR = 1.016). Herein, Cluster C1 gene expression was mostly associated with changes in eHCC development. Our results indicate selected prognostic signature DEGs relevant to the tumor type stratification in HCC from TCGA, where tumors with higher rates of these Cluster C1 dysregulation may be aggressive than those of Cluster C3 determined phenotype.

### Construction of the Prognostic Gene Signature and Immune Functional Annotation

**Figure 4:**
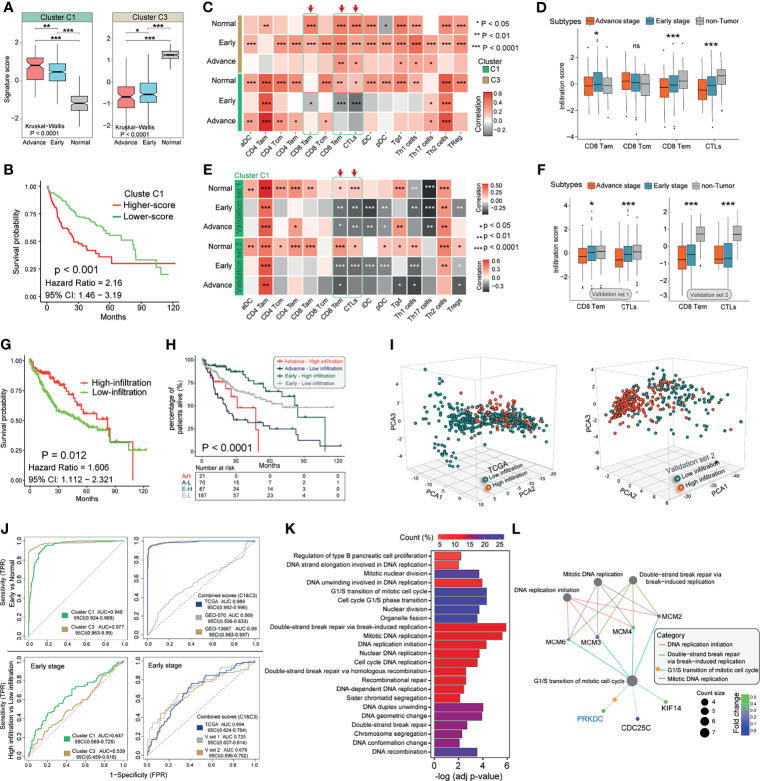
Characteristics of Cluster signatures in HCC immune environment and biological functions. **(A)** Distribution of Cluster C1 and Cluster C3 signature scores in groups with different pathological stages (aHCC, eHCC, and non-tumor). The differences among groups were compared through the Kruskal–Wallis test (Kruskal–Wallis, *p* < 0.001). **(B)** Kaplan–Meier survival analyses of Cluster C1 signatures scores (log-rank test, *p* < 0.001). **(C)** Correlations between Cluster C1/C3 signatures and 15 types of immune cell in different pathological stages patients from TCGA. Negative correlation was marked with gray and positive correlation with orange. Red arrow indicates the potential immune cells associated with Cluster C1 signature. **(D)** The infiltration of CD8-Tam/Tcm/Tem and CTL immune cells in three HCC groups from TCGA. Within each group, the scattered dots represent immune cell prediction values. The lines in the boxes represent median value. The statistical difference of three groups was compared through the one-way ANOVA. **(E)** Correlations between Cluster C1 signature and 15 types of immune cells in different pathological stage patients form GEO. Data from validation set 1 (upper) and set 2 (lower) platform were used. **(F)** The infiltration of CD8-Tem and CTL immune cells in three HCC groups from GEO. Data from validation set 1 (left) and set 2 (right) platform were used. **(G)** Kaplan–Meier survival analysis of CD8-Tem/CTL-based immune infiltration levels (log-rank test, *p* = 0.012). Red, high infiltration; green, low infiltration. **(H)** Kaplan–Meier curves for patients with eHCC/aHCC in TCGA cohort stratified by CD8-Tem/CTL-based immune infiltration. Log-rank test shows an overall *p* < 0.0001. **(I)** Principal component analysis (PCA) for the transcriptome profiles of Cluster C1 genes in different immune infiltration patterns from eHCC and non-tumor samples. **(J)** ROC curves measuring the predictive value of Cluster C1, Cluster C3 (left), and combination of Cluster C1 and C3 (right) in eHCC diagnosis and CD8-Tem/CTL-related infiltration. **(K)** Functional annotation for Cluster C1 genes between different infiltration groups in eHCC patients. The color depth of the barplots represents the number of genes enriched. **(L)** Subnetworks that contain higher selective marker-regulated nodes implicated in the biological functions. The grade of the color represents the expression level of genes. Count size indicates the nodes enriched in each category. *p < 0.05; **p < 0.01; ***p < 0.0001; ns, no significant difference. HCC, hepatocellular carcinoma; aHCC, advanced-stage hepatocellular carcinoma; eHCC, early-stage hepatocellular carcinoma; TCGA, The Cancer Genome Atlas; CTL, cytotoxic T cell; GEO, Gene Expression Omnibus.

To identify the underlying biological characteristics of these prognostic gene modification phenotypes in the eHCC, we fix our attention on TCGA cohort and validated results in two GEO cohorts, which comprised more than 590 eHCC, 210 aHCC, and 600 non-tumor cases and offered the most comprehensive functional annotation. There were significant distinct patterns of Cluster C1 and Cluster C3 signature in three proposed subtypes of TCGA and consistent with the above outcomes (**Figure 4A**). Higher Cluster C1 signature score was associated with poor overall OS (**Figure 4B**). The stratification between non-tumor, eHCC, and aHCC was significantly accompanied with decreased Cluster C1 and increased Cluster C3 signature score. Then, we evaluated the association between two clusters and T cell-related immune cells infiltration from transcriptomic data in both TCGA and GEO validation sets. The eHCC patients were characterized by the type of dysregulated immune cells and presented variable association with different cluster types; Cluster C1 signature showed negative association with activated CD8 T cell (CD8 Tam), Effector memory CD8 T cell (CD8 Tem), and cytotoxic T cell (CTL) infiltration levels in eHCC and aHCC groups and simultaneously presented positive correlation in non-tumor group; Cluster C3 signature showed positive association with CD8 Tam, CD8 Tem, and CTL infiltration levels in eHCC, aHCC, and non-tumor groups (**Figure 4C**). In addition, differences in clinical subgroups of HCC were assessed in TCGA series, and a lower CD8 Tem and CTLs level was significantly associated with tumor development from normal to aHCC (**Figure 4D**). Consistent with these findings, the correlation analyses between Cluster C1 signature and CD8 Tem and CTLs in two GEO validation cohorts also showed an inspiring result (**Figure 4E**), and the CD8 Tem and CTLs also negatively associated with HCC development in two independent cohorts (**Figure 4F**).

To further characterize and understand the immune cell clinical differences among these HCC patients, we proposed subdividing tumor into two subtypes as high-infiltration group and low-infiltration group. Differences in the CD8 Tem- and CTL-based molecular subtypes were evaluated in TCGA cohort, and lower infiltration in HCC was significantly associated with poor prognosis (HR, 1.606; 95% CI: 1.112–2.321; *p* = 0.002; **Figure 4G**). The specific immune cell infiltration was also investigated in between eHCC and aHCC patients to explore whether the association of immune disorder affected the ability of Cluster C1 to predict the eHCC and survival outcomes, and the survival shortcoming of the low infiltration in both patients who suffer from eHCC and aHCC was the most obvious (log-rank test; *p* < 0.0001) (**Figure 4H**). In addition, within the mRNA expression of the eHCC and non-tumor samples, we used CD8 Tem- and CTL-related high/low infiltration as a pattern recognition variable. Based on 13 Cluster C1 members from TCGA and 25 Cluster C1 members from GEO validation set 2, the clustering and changing trends of each sample were visually displayed on the PCA map (**Figure 4I**). Despite individual variability, the graphics show appreciable separation of infiltration condition between two cohorts.

We next interrogated TCGA and GEO validation cohorts’ prediction value with Cluster C1 and Cluster C3 signature. We evaluated the diagnostic performance of two clusters in discriminating the eHCC from non-tumor group. In TCGA cohort, the analysis demonstrated that Cluster C1 (AUC = 0.946; 95% CI: 0.924–0.968) and Cluster C3 (AUC = 0.977; 95% CI: 0.963–0.99) signature possessed a high accuracy in predicting eHCC (**Figure 4J**, upper left). Moreover, combining Cluster C1 and Cluster C3 signatures improved the predictive value compared with that of Cluster C1 or Cluster C3 alone in both TCGA and GEO validation set 2 (**Figure 4J**, upper right). We then evaluated the predictive value of Cluster C1 and Cluster C3 signature in TCGA eHCC group; and the predictive value of Cluster C1 (AUC = 0.647; 95% CI: 0.569–0.725) to CD8 Tem- and CTL-related immune infiltration was also confirmed (**Figure 4J**, lower left). Meanwhile, combining Cluster C1 and Cluster C3 slightly improved the predictive value and presented a similar tendency in three independent cohorts (**Figure 4J**, lower right). Moreover, GO enrichment analysis of Cluster C1/C3 signature gene function in eHCC immune subgroups (TCGA) was conducted using the R package clusterProfiler, which was used to discover the potential regulatory relationships among these signature genes in biological functions. The BPs with significant enrichment are summarized in **Table S6**. These Cluster genes showed distinct BPs between high- and low-infiltration groups (**Figure 4K**), especially in cell cycling and proliferation regulation in eHCC TME. Surprisingly, the PRKDC showed enrichment of BPs remarkably related to transition of mitotic cell cycle and DNA replication and break repairing-related MCM family member genes (**Figure 4L**). Consistent with **Figure 2A** of gene mutation frequency, the cell regulation potential confirmed again that PRKDC played a non-negligible role in the eHCC TME. These findings could demonstrate that Cluster C1 signature and PRKDC modification patterns potentially predict the eHCC and tumor immune microenvironment formation.

### Association of PRKDC Dysregulation and Immune-Related Prognosis Risk

**Figure 5:**
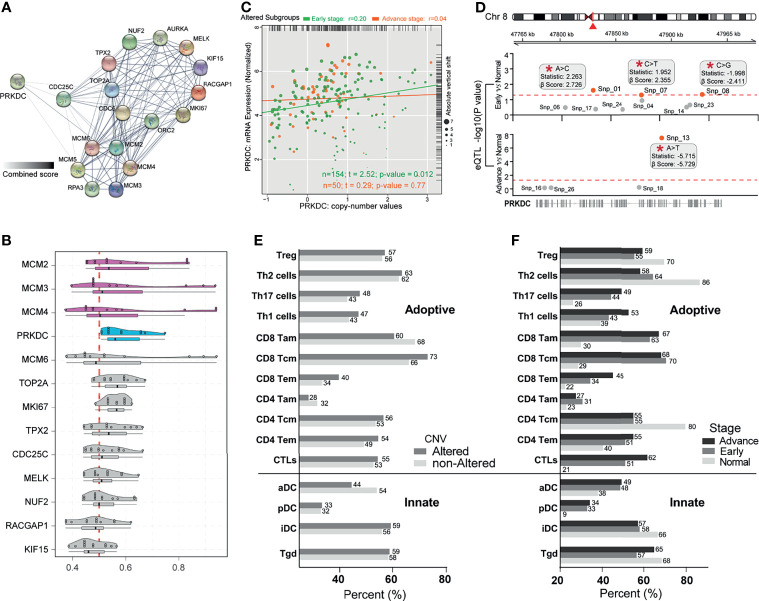
Identification of PRKDC dysregulation in eHCC and immune-related poor prognostic signatures. **(A)** The PPI network analysis of Cluster C1-related differentially expressed proteins. Thicker lines and deeper color represent the closer association between each other. **(B)** Summary of functional similarities of Cluster C1 genes. The distributions of functional similarities and ranked central proteins are summarized as boxplots; lines in the boxes indicate the mean of average value. **(C)** Scatter plots depicting the positive correlation between PRKDC expression and copy number variation in patients with eHCC and aHCC. Pearson’s correlation coefficient is shown in the graphs. **(D)** Cis-eQTLs for PRKDC in two subgroups of HCC patients. Significant novel SNPs are indicated by the red mark and labels (*). The statistics used is correlation coefficient “r”; and β-Score is the confidence index. The red line corresponds with a threshold of *p* ≤ 0.05. **(E)** Immune cell-related poor prognostic signature profiles. Comparison of immune cell-related poor prognostic signature profiles in HCC patients with altered PRKDC (n = 204) *versus* non-altered PRKDC (n = 161). Alteration including somatic mutation and copy number amplification, gain, and shallow deletion. Gray bars indicate the percentage of patients having immune cell-related poor prognostic signature in altered-PRKDC HCC group, while the gray bars represent the percentage in non-altered PRKDC group. **(F)** Comparison of immune cell-related poor prognostic signature profiles in three pathological stage patients: eHCC (n = 91), aHCC (n = 274), and non-tumor (n = 161). Black bars represent the percentage of aHCC patients, gray bars represent the percentage of eHCC patients, and gray bars represent the percentage of non-tumor samples. Immune cell signatures were classified to adaptive and innate. eHCC, early-stage hepatocellular carcinoma; PPI, protein–protein interaction; aHCC, advanced-stage hepatocellular carcinoma; eQTL, expression quantitative trait locus; SNP, single-nucleotide polymorphism.

Interactomics holds great promise in understanding the molecular mechanism of cells affected by biological factors. To examine Cluster C1-related proteins and their protein–protein interaction (PPI), the STRING database (53) was used to deduce enriched proteins and generated a PPI network (**Figure 5A**). The PPI network depicted functional attributes of PRKDC to Cluster C1-related proteins, including CDC25C, MCM4, and TOP2A. To further identify the essential of PRKDC interactome in eHCC, we ranked Cluster C1 members by their average functional similarity relationships within the interactome (54). The MCM2/3/4 and PRKDC (cutoff value >0.54) were two types of top-ranked proteins potentially playing central roles in Cluster C1 (**Figure 5B**). PRKDC, which has not yet been previously identified as an important partner of eHCC, has been previously reported to play an important role in HCC (55) and T cell-related immunodeficiency (56). As the PRKDC possessed the highest average functional similarity in our analyses, it is eligible for further investigation.

Previous studies have identified that PRKDC genetic alteration is associated with gene expression signature and influenced immune cell-related immunodeficiency (24, 55–57). To determine oncogenic events across the different subgroups, we investigated the indels and CNVs affecting gene expression in eHCC. There was a moderate correlation of the expression of PRKDC genes with copy number-altered values in TCGA cohort, which presented a higher correlation in eHCC (Spearman’s correlation rho = 0.2, *p* = 0.012) compared with aHCC (Spearman’s correlation rho = 0.04, *p* = 0.77) (**Figure 5C**). Meanwhile, we noted a significant increase of PRKDC expression between eHCC and non-tumor samples, which was associated with single-nucleotide polymorphism (SNP). The eQTL analysis observed that snp_01 site (statistics r: 2.263; β-Score: 2.726) and snp_07 site (statistics r: 1.952; β-Score: 2.355) were positively associated with PRKDC expression in eHCC (**Figure 5D**). Furthermore, poor prognostic signature analysis based on the obtained different immune cells profile in adaptive and innate immunity, as well as pathological stages and PRKDC CNVs, was examined in TCGA. Each type of immune cell corresponding maximum rank survival statistic was selected as a poor prognostic indicator and dichotomized the HCC and non-tumor samples. In TCGA cohort, the CD8 Tem (40% vs. 34%) and CTLs (55% vs. 53%) related risk frequencies were higher in the PRKDC genetic altered group of HCC patients (**Figure 5E**). Concurrently, the CD8 Tem (non: 22%, early: 34%, advanced: 45%) and CTLs (non: 21%, early: 51%, advanced: 62%) were observed to have lower risk frequencies in non-tumor and eHCC, compared with aHCC (**Figure 5F**).

### Evaluation of Gene-Set Signature and PRKDC in Different Hepatocellular Carcinoma Rats

**Figure 6:**
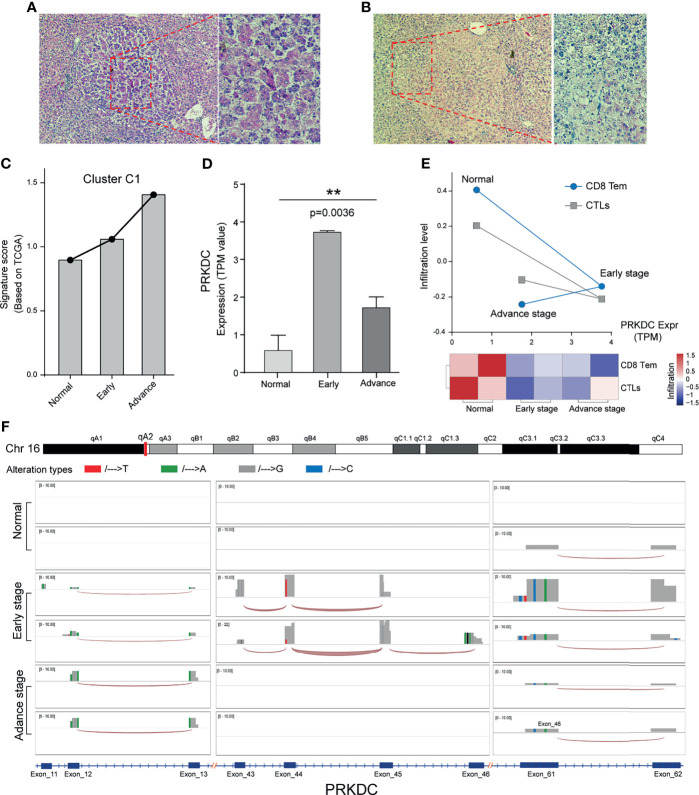
Cluster C1 signature and PRKDC characteristics of HCC *in vivo*. **(A, B)** Liver tissue of aHCC rats; H&E staining demonstrates clear lesions in hepatic nodules, composed of pleomorphic cells with prominent glandular and trabecular formation, as well as eosinophilic focus of cellular alteration with pale pink cytoplasm. **(C)** Relative Cluster C1 signature quantification in DEN-induced eHCC rat model, compared with aHCC and normal. TPM value was applied for gene expression comparison. **(D)** Relative mRNA quantification of PRKDC in DEN-induced eHCC rat model. TPM, transcripts per million reads. ** indicates *p* = 0.0032. **(E)** Evaluation of CD8 Tem and CTL infiltration level and relationship to PRKDC in eHCC rat model. Red represents immune status, and blue represents infiltration grades. **(F)** Landscape of PRKDC genomic map in three groups of HCC rat models. Three high-frequency nucleotide variation regions of PRKDC were selected to be displayed in the windows. Different alteration tendencies of A/T/G/C were colored with red, green, gray, and blue. The height of the bar represents the read count number. The splice junction track between the different exon was connected by an arc. HCC, hepatocellular carcinoma; aHCC, advanced-stage hepatocellular carcinoma; DEN, diethylnitrosamine; eHCC, early-stage hepatocellular carcinoma; CTL, cytotoxic T cell.

Next, we used the DEN-induced rat eHCC to test if Cluster C1 and PRKDC play prior roles in tumorigenesis. The application of DEN has an irreversible carcinogenic effect in rodents (58). In addition, repeated injection of low-dose 50 mg/kg DEN could generate the disease that more closely resembles the human pathology. H&E staining demonstrated a clear morphological change of eHCC and aHCC, compared with normal (**Figures 6A, B**; **Figure S2**). Overall, we observed that Cluster C1 from TCGA presented a higher signature score in eHCC and aHCC groups compared with control group, which presented an adverse effect on human health and accelerates HCC malignant behaviors (**Figure 6C**). In addition, the higher level of PRKDC can be significantly detected in eHCC/aHCC compared with normal rat tissue (*p* = 0.0036) (**Figure 6D**). The immune characteristics and dysregulation of biomarker gene expression are very common and typically have a profound impact on the TME. Unexpectedly, our result found that the CD8 Tem and CTL cell infiltration level were obviously decreased in the eHCC/aHCC rat groups and negatively associated with PRKDC expression (**Figure 6E**). In the context of PRKDC dysregulation, structural alteration results in their genomic mutation and substantial tumor-regulating roles in eHCC pathogenesis. In support of this hypothesis, evaluating the mRNA transcripts discovered read count amplification and nucleotide alterations in the three exon regions of eHCC/aHCC compared with normal rat tissues (**Figure 6F**). The splice junction track shows the exon interaction in the genomic landscape map. The above results, in line with our prediction in public datasets, confirm that, in the eHCC development, unsupervised Cluster C1 signature and PRKDC dysregulation were important predictors and associated with CD8 Tem and CTL characteristics in TME.

## Discussion

In the present study, we showed increased gene expression and mutation in eHCC in association with tumorigenesis and immune milieu, supporting dysregulated specific metagene and immune cells as potential mechanism and predictors. Having demonstrated the tumor-specific DEGs of Cluster C1, the prior role of PRKDC was found to be associated with CD8 Tem and CTL infiltration levels in eHCC. Of note, we observed that increased Cluster C1 signature and decreased CD8 Tem and CTLs were both independent poor prognostic factors for survival in HCC patients. Due to the absence of specific symptoms in eHCC and the lack of early diagnostic markers, most patients with HCC are often diagnosed in an advanced stage with poor prognosis (59, 60), identifying that the characteristics of HCC initiation and development in the genomics and immune environment will contribute to enhancing our understanding of novel diagnostic markers for eHCC and TME pattern and guiding more effective immunotherapy strategies.

The role of the tumor field effect of genomic instability and oncogene overexpression in HCC has gained much interest in recent years (61–63), and currently an altered TME is considered a promoter of cancer (64, 65). Although under physiologic conditions immune disorder is an adaptive response to genetic alteration (66, 67), when the immune disorder stimuli persist, the non-resolved immunodeficiency contributes to carcinogenesis (15, 68). Along with these lines, the concept of genetic alteration and tumor immune microenvironment, such as TP53/GATA4 mutation, CXCL10 expression, and infiltrating immune cells (monocytes, T, B, and NK cells), has been previously associated with cancerization in the liver (61, 69, 70). With this study, we provide a comprehensive description of a diagnostic signature and immune microenvironment characteristics underlying the eHCC. To this end, we first identified 414 upregulated and 272 downregulated DEGs related to HCC development, as well as constructing mutational significance with eligible sample to define new biologically and clinically relevant genes not previously appreciated. From the 77 DEGs that were shown to have a higher mutation level and an association with OS, 53 feature genes were further screened. The analysis showed that PRKDC possessed the highest mutation frequency in the eHCC group compared with the aHCC group, which also significantly associated with poor OS in HCC patients (HR = 1.79, 95% CI: 1.26–2.53). This finding is in line with previous reports suggesting that PRKDC mutation was closely connected to various tumors (24). However, integrated analysis of these feature gene expression revealed the difficult to stratify the pathological stage and functional annotation in the HCC patients. At the same time, we recognize that the more rigorous approach should be used to split the data into different groups with acceptable statistical power (71, 72).

The heterogeneity in HCC gives rise to distinct tumor subclasses based on environmental factors, genetic heterogeneity, inflammation, and immune infiltration (73–76), leading to a growing interest into translating this information into clinical practice for HCC treatment and prediction, as well as developing the personalized therapies based on unique intrinsic molecular signatures. To identify the most promising candidates for eHCC diagnosis, we conducted the patient-based unsupervised analysis using a compendium of feature gene sets recapitulating the tumor’s specific molecular signature in three independent cohorts. The association between poor and favorable prognosis DEG expression and expression level of Cluster C1/C3 genes provides a biological significance to HCC stratification and supports targeting tumor specific markers as a mean to reprogram an aggressive tumor type. Importantly, Cluster C1 (upregulated DEGs) expression showed a better ability in distinguishing HCC stratification, which is significantly associated with the shortest OS in aHCC/eHCC subgroups and poor prognosis in aHCC compared with eHCC. Meanwhile, the survival benefit associated with Cluster C3 expression (downregulated DEGs) could be an indirect evidence to support the prior role of Cluster C1. Therefore, the correlation of Cluster C1 expression and prognostic subtypes corroborated the role of unique molecular signatures in tumor development in HCC.

Emerging data support the idea that the TME cells play a crucial role in liver cancerization, HCC development, chemoresistance, and recurrence (15, 77–80). Here, we revealed a comprehensive landscape of crosstalk between the specific prognostic clusters, clinical characteristics of HCC, and immune cell infiltration. With the help of several computational algorithms, integrated analysis revealed that Cluster C1 not merely act as a prognostic biomarker for eHCC but also significantly associated with immune cell dysregulation in patients of different clinical subtypes. Patients with a lower level of CD8 Tem and CTLs and presented immunosuppressive nature of HCC TME and reduced protection against external stimulus (81–83) were significantly related to Cluster C1 signature scores in eHCC. Moreover, lower T lymphocyte infiltration in HCC was previously reported to associate with innate immunosuppression and tumor mutation burdens, such as Tregs, cytokines (TGF-β and IL-10), and marker gene mutation frequency (84–86). Considering the changes in the TME between non-tumor and HCC (87, 88), our Cluster C1 signature has shown a predictive advantage in distinguishing the specific eHCC immune cell (CD8 Tem and CTLs) infiltration level from non-tumor samples. In this respect, in line with previous studies (89–92), these two immune cells (CD8 Tem and CTLs) markedly elucidated the immune characteristics of HCC initiation and progress, which has also shown benefit in improving patient prognosis in both eHCC and aHCC. Therefore, we infer that the effect of Cluster C1 signature on the eHCC patients is probably related to the remodeling of specific immune cells in the TME. By applying ROC curve analysis (47), we also demonstrated the predictive value of Cluster C1 signature for the liver cancerization and the CD8 Tem/CTL-based immune infiltration in three separate cohorts of patients with eHCC. Of note, combining Cluster C1 and C3 signaling can slightly improve the prediction accuracy, although diagnostic accuracy of Cluster C3 signature alone was not acceptable. Taken together, these results provide new insights for immune cell omics research on the mechanism by specific genes regulating the survival of eHCC patients.

To explore potential therapeutic target mechanism for HCC patients with poor immune infiltration, we further performed biological functional analysis using gene expression data from TCGA. The result of Cluster C1 gene-set showed that the core molecular PRKDC and its associated genes were significantly correlated with the cell cycling and DNA replication. Previous studies indicated that both cell cycling and DNA replication impairment were related to T-cell inhibition and tumor cell death (93–95). Furthermore, our verification in TCGA-HCC patients confirmed that PRKDC dysregulation was mostly associated with its genomic instability, especially in eHCC patients. In addition to the transcriptional regulation, SNPs in eHCC are also significant cis-eQTLs for the PRKDC expression. The SNP locus in cancer was demonstrated to influence the checkpoint gene-related immune disorder and target gene expression (96, 97), suggesting that the locus variation has important role link between gene expression and tumorigenesis. At present, the PRKDC heterozygous mutation has been reported to impair the DNA double-strand break (DSB) repair and contribute to immunodeficiency (57). Not surprisingly, the PRKDC genetic alteration is emerging as a predictive biomarker and drug target for anti-tumor immunotherapy in various malignancies (24). The PRKDC mutation in patients exhibited a skewed cytokine response typical of Th2 and Th1 cells (56) and influenced the immune responses (98). Moreover, higher PRKDC mutation and expression were correlated with ER^−^ breast cancer immune pathway functions (99). In HCC, PRKDC expression was proved to be associated with shorter OS and immune cell infiltration (100). Our finding is interesting given the important role of PRKDC in specific immune cells (CD8 Tem and CTL)-related poor survival rate in the context of elevated CNV in HCC patients. Consistent with this, for CD8 Tem and CTLs, the lower prognostic frequencies suggested the immune cells’ clinical effects in initiation and progression of HCC. On the other hand, in DEN-induced eHCC rat model, our experimental verification confirmed that both Cluster C1 signature and PRKDC expression were shown to be positively associated with tumorigenesis, as well as downregulated CD8 Tem and CTL infiltration level. In principle, somatic mutation shows its primary effects on the expression of cancer-relevant genes in tumorigenesis, indicating that it is a powerful driver of intratumoral heterogeneity and progression (101). Thus, the evaluation of PRKDC genomic instability and expression to enable a better understanding of tumorigenesis is an effort to provide fresh and novel insights for developing a biomarker in combination with bioinformatics prediction. Taken together, the preliminary findings suggest a diversity in HCC TME, offering a comprehensive view of the relative level of immune subtypes and providing insights about the crosstalk between specific target genes, eHCC, and immune characteristics.

## Conclusion

In conclusion, we identified a gene set-based prognostic signature using a large number of individuals and effectively differentiate the eHCC from aHCC and non-tumor controls with a high accuracy. Our study demonstrated that the eHCC was characterized by specific immune cell disorder, namely, CD8 Tem and CTLs, both of which were closely associated with Cluster C1 signature. Of note, given the correlation among the genome instability, PRKDC expression, and immune cell-related poor prognostic signature, PRKDC can be a potential candidate to HCC patients’ early diagnosis and selection for immunotherapy. These findings have implications in specific gene-signature and tumor immune environment characteristics in HCC patient stratification and could be of benefit in developing novel immunotherapies.

## Data Availability Statement

All the original data of the current study are available from the corresponding author on reasonable request. The public available datasets were summarized in the **Table S7**. The rat RNA-seq data is available from SRA hub, Bioproject number is PRJNA772097 (https://www.ncbi.nlm.nih.gov/sra/?term=PRJNA772097).

## Ethics Statement

All animal experiments were approved by the Animal Ethics Committee of Chiang Mai University, Thailand (Protocol Number: 2563/RT-0015).

## Author Contributions

QZ: conceptualization, data curation, formal analysis, and writing—original draft. AC: methodology (animal experiments). RW: methodology (animal experiments). ZX: conceptualization, writing—review and editing, and supervision. CP: conceptualization, methodology (animal experiments), writing—review and editing, supervision, and funding acquisition. All authors contributed to the article and approved the submitted version.

## Funding

This study was funded by grants from the National Research Council of Thailand (NRCT) and Faculty of Medicine Research Fund, Chiang Mai University (Grant No. 142/2563).

## Conflict of Interest

The authors declare that the research was conducted in the absence of any commercial or financial relationships that could be construed as a potential conflict of interest.

## Publisher’s Note

All claims expressed in this article are solely those of the authors and do not necessarily represent those of their affiliated organizations, or those of the publisher, the editors and the reviewers. Any product that may be evaluated in this article, or claim that may be made by its manufacturer, is not guaranteed or endorsed by the publisher.

## Supplementary Material

Supplementary Figure 1
**(A)**. Heatmap depicting consensus clustering solution (k = 3) for significant prognostic DEGs (poor/favor) in the advance and early diagnostic HCC patients (n = 365). Scatter plot showing median expression levels of poor-prognosis (x-axis) gene and favor-prognosis gene (y-axis) in each HCC patients. Signature subgroups were assigned based on the relative expression levels of selected Cluster C1 and Cluster C3 genes. Heatmap depicting expression levels of Cluster C1 and Cluster C3 genes across each subgroup in different pathological stages. **(B)**, Heatmap depicting consensus clustering solution (k = 3) for significant prognostic DEGs (poor/favor) in the advance and early diagnostic HCC patients (n = 365). Scatter plot showing median expression levels of poor-prognosis (x-axis) gene and favor-prognosis gene (y-axis) in each HCC patients. Signature subgroups were assigned based on the relative expression levels of selected Cluster C1 and Cluster C3 genes. Heatmap depicting expression levels of Cluster C1 and Cluster C3 genes across each subgroup in different pathological stages. **(C)**. Overlap of cluster C1 and C3 genes in TCGA and two GEO datasets.

Supplementary Figure 2 Histopathological changes in the livers from DEN induced rats and control rat. **(A)**, liver tissue from control rat; H&E staining revealed normal cellular architecture where hepatocytes are arranged in cell plates separated by sinusoids rat, **(B)**, Liver tissue of eHCC rat; H&E staining demonstrated eosinophilic focus of cellular alteration (left) including small cell change (middle), and clear cell focus of cellular alteration (right).

Supplementary Table 1 A total genes between tumor and normal with available clinical information.

Supplementary Table 2 Significantly expressed genes.

Supplementary Table 3 Overview of 53 differently expressed genes detail.

Supplementary Table 4 Enrichment of DEGs in different stage HCC patients.

Supplementary Table 5 Fisher’s test of the cluster subtype.

Supplementary Table 6 Biological processes

Supplementary Table 7 Summary of publicly available datasets used for this study.
