# Supplementary figures and images for: Identification of Gene-Set Signature in Early-Stage Hepatocellular Carcinoma and Relevant Immune Characteristics
Source: Front Oncol. 2021 Oct 22;11:740484. doi: 10.3389/fonc.2021.740484 (PMC8570321; doi:10.3389/fonc.2021.740484)

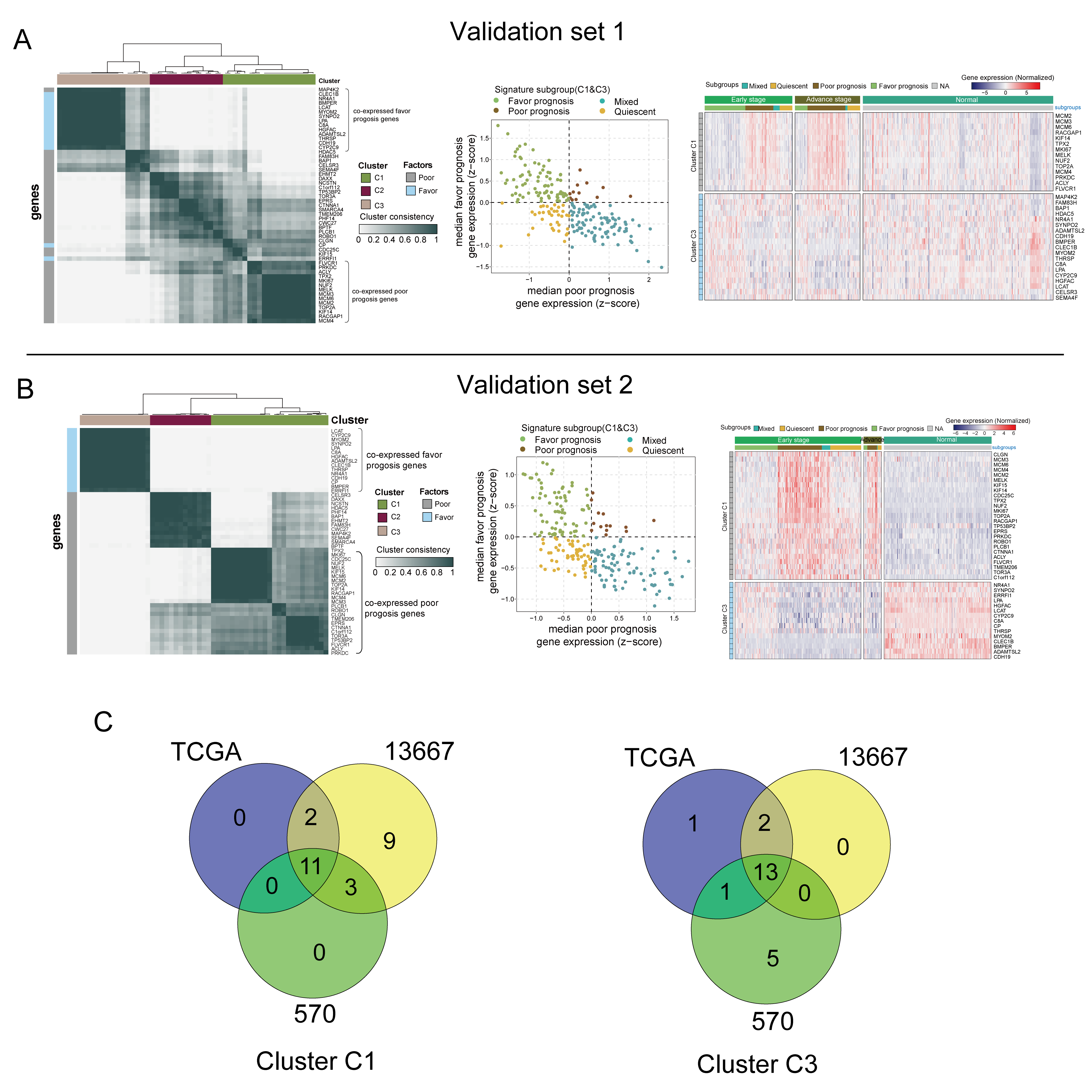

Supplement: Supplementary Figure 1 — (A). Heatmap depicting consensus clustering solution (k = 3) for significant prognostic DEGs (poor/favor) in the advance and early diagnostic HCC patients (n = 365). Scatter plot showing median expression levels of poor-prognosis (x-axis) gene and favor-prognosis gene (y-axis) in each HCC patients. Signature subgroups were assigned based on the relative expression levels of selected Cluster C1 and Cluster C3 genes. Heatmap depicting expression levels of Cluster C1 and Cluster C3 genes across each subgroup in different pathological stages. (B), Heatmap depicting consensus clustering solution (k = 3) for significant prognostic DEGs (poor/favor) in the advance and early diagnostic HCC patients (n = 365). Scatter plot showing median expression levels of poor-prognosis (x-axis) gene and favor-prognosis gene (y-axis) in each HCC patients. Signature subgroups were assigned based on the relative expression levels of selected Cluster C1 and Cluster C3 genes. Heatmap depicting expression levels of Cluster C1 and Cluster C3 genes across each subgroup in different pathological stages. (C). Overlap of cluster C1 and C3 genes in TCGA and two GEO datasets. [file Image_1.tif]

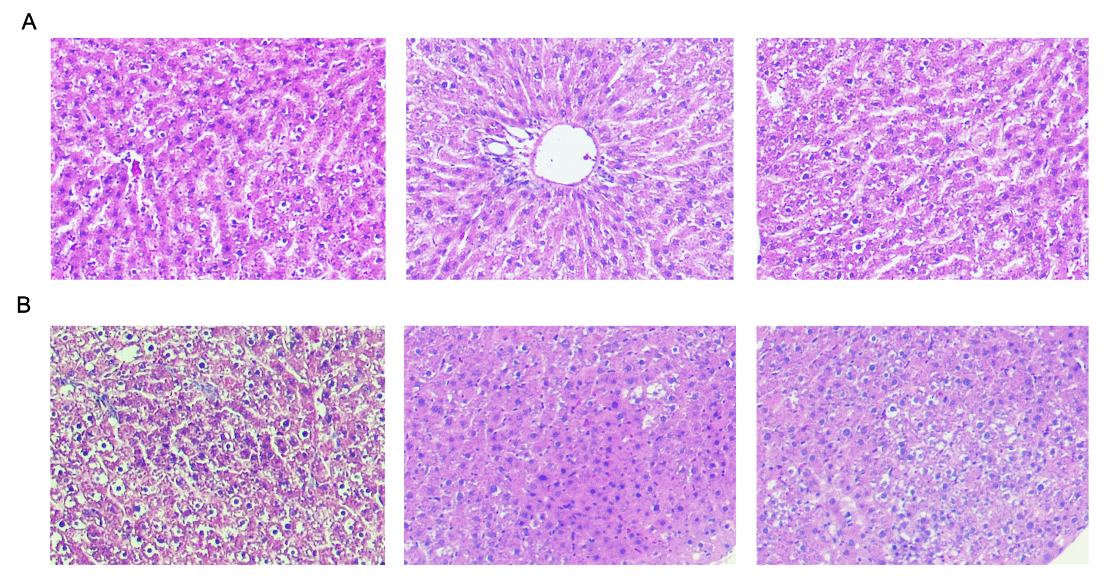

Supplement: Supplementary Figure 2 — Histopathological changes in the livers from DEN induced rats and control rat. (A), liver tissue from control rat; H&E staining revealed normal cellular architecture where hepatocytes are arranged in cell plates separated by sinusoids rat, (B), Liver tissue of eHCC rat; H&E staining demonstrated eosinophilic focus of cellular alteration (left) including small cell change (middle), and clear cell focus of cellular alteration (right). [file Image_2.tif]
